# Prognostic significance of papillary muscle infarction detected by late gadolinium-enhanced MRI in acute reperfused ST-segment elevation myocardial infarction

**DOI:** 10.1186/1532-429X-15-S1-P253

**Published:** 2013-01-30

**Authors:** Ingo Eitel, Dörthe Gehmlich, Henning Sünkel, Josefine Meissner, Suzanne de Waha, Steffen Desch, Georg Fuernau, Matthias Gutberlet, Gerhard Schuler, Holger Thiele

**Affiliations:** 1 Cardiology, Heart Center Leipzig, Leipzig, Germany; 2 Radiology, University of Leipzig - Heart Center, Leipzig, Germany

## Background

The frequency and prognostic significance of papillary muscle infarction (PapMI) have not been investigated in a large multicenter study of patients with acute reperfused ST-elevation myocardial infarction (STEMI) because papillary muscle necrosis is difficult to visualize premortem. Noninvasive investigation by late gadolinium-enhanced magnetic resonance imaging (LGE-MRI) enables the detection of PapMI with high tissue contrast and spatial resolution. Aim of the study was to assess the exact frequency and clinical characteristics of PapMI using LGE-MRI in patients with STEMI.

## Methods

We enrolled 795 STEMI patients reperfused by primary percutaneous coronary intervention (<12 h after symptom onset) in this CMR study at 8 centers. The CMR was completed within one week after infarction using a standardized CMR protocol. Central core lab-masked analyses for the presence of PapMI were performed. The primary clinical end point of the study was the occurrence of major adverse cardiovascular events (MACE) defined as death, reinfarction, and congestive heart failure within 12 months after infarction.

## Results

PapMIs were detected in 12% of our patients. The posterior papillary muscle was involved more frequently than the anterior papillary muscle (68% versus 16%; P<0.001), whereas in 18% both PapMIs (anterior and posterior) were infarcted. PapMI was encountered more frequently in patients with right coronary artery and left circumflex lesions compared with left anterior descending artery lesion (50%, 39% and 11%, P<0.001). The presence of PapMI was associated with larger infarcts (p=0.01), less myocardial salvage (p<0.001), and impaired left ventricular function (P=0.001). By multiple logistic regression analysis, PapMI was identified as an independent predictor of the presence of MACE (p<0.001).

## Conclusions

PapMI is a frequent complication in patients with acute reperfused STEMI associated with larger infarcts, less myocardial salvage, impaired left ventricular function and adverse clinical outcomes.

## Funding

None

